# 2385 Role of the antioxidant enzyme catalase in respiratory syncytial virus infection

**DOI:** 10.1017/cts.2018.118

**Published:** 2018-11-21

**Authors:** Maria Ansar, Jeffrey M. Chambliss, Naryana Komaravelli, Teodora Ivanciuc, Antonella Casola, Roberto P. Garofalo

**Affiliations:** 1 University of Texas Medical Branch, Galveston, TX, USA; 2 Department of Pediatrics, University of Texas Medical Branch, Galveston, TX, USA

## Abstract

OBJECTIVES/SPECIFIC AIMS: The goal of this study is to further evaluate underlying disease parameters in respiratory syncytial virus (RSV) infection, that is reduction in antioxidant potential, and determining if supplementation of the antioxidant enzyme catalase could be employed as a potential therapeutic. METHODS/STUDY POPULATION: Nasopharyngeal secretions were obtained from patients (<2 years old) verified for RSV infection, and assessed for catalase activity and correlated with disease parameters. In addition, the BALB/c animal model of RSV infection was utilized to directly study the effect of supplemental catalase on RSV-related disease parameters in vivo. The catalase formulation used in these studies is pegylated, and has been tested to provide long-term increased catalase activity in vivo. We are also currently working on designing an in vitro model of catalase supplementation in A549 bronchial epithelial cells. RESULTS/ANTICIPATED RESULTS: Our preliminary data shows that patients with more severe disease (based on hospitalization, oxygen supplementation) have significantly lower levels of catalase activity (*p*<0.02). Additionally, when pegylated-Catalase (PG-CAT) treatment is utilized in RSV infection of mice, there is significant improvement in several disease parameters. PG-CAT-treated mice show an attenuated body weight loss (*p*<0.001) and clinical disease (*p*<0.02), and also have lower levels of key pro-inflammatory cytokines including CXCL1 and TNF-α. PG-CAT treatment also resulted in a minor decrease in viral titer, which is being further evaluated. In addition, PG-CAT treatment resulted in an improvement in airway hyperresponsiveness observed at baseline, we are further characterizing this improvement and also conducting methacholine challenges. Currently, we are working to determine the underlying mechanism through which PG-CAT results in these improvements, and whether it is through changes in immune cell populations, cellular signaling or apoptosis signaling pathways (i.e., caspases). DISCUSSION/SIGNIFICANCE OF IMPACT: RSV is the leading cause of viral pneumonia and bronchiolitis in infants, with no vaccines or effective therapeutics available currently. Our study indicates that catalase activity could be used as a potential correlate for disease severity and be used as an indicator of disease during patient treatment. Additionally, and more importantly supplementation of catalase could be used as a potential therapeutic for treatment of RSV.

